# Identifying the optimal regional predictor of right ventricular global function: a high‐resolution three‐dimensional cardiac magnetic resonance study†

**DOI:** 10.1111/anae.14494

**Published:** 2018-11-14

**Authors:** T. J. W. Dawes, A. de Marvao, W. Shi, D. Rueckert, S. A. Cook, D. P. O'Regan

**Affiliations:** ^1^ National Heart and Lung Institute Imperial College London London UK; ^2^ Medical Research Council London Institute of Medical Sciences Faculty of Medicine Imperial College London London UK; ^3^ Department of Computing Faculty of Engineering Imperial College London London UK; ^4^ Department of Clinical and Molecular Cardiology Medical Research Council London Institute of Medical Sciences Faculty of Medicine Imperial College London London UK; ^5^ Department of Cardiology National Heart Centre Singapore Singapore and Duke‐NUS Graduate Medical School Singapore; ^6^ Medical Research Council London Institute of Medical Sciences Faculty of Medicine Imperial College London London UK

**Keywords:** cardiac morbidity: pre‐operative factors, magnetic resonance imaging, right ventricular function

## Abstract

Right ventricular (RV) function has prognostic value in acute, chronic and peri‐operative disease, although the complex RV contractile pattern makes rapid assessment difficult. Several two‐dimensional (2D) regional measures estimate RV function, however the optimal measure is not known. High‐resolution three‐dimensional (3D) cardiac magnetic resonance cine imaging was acquired in 300 healthy volunteers and a computational model of RV motion created. Points where regional function was significantly associated with global function were identified and a 2D, optimised single‐point marker (SPM‐O) of global function developed. This marker was prospectively compared with tricuspid annular plane systolic excursion (TAPSE), septum‐freewall displacement (SFD) and their fractional change (TAPSE‐F, SFD‐F) in a test cohort of 300 patients in the prediction of RV ejection fraction. RV ejection fraction was significantly associated with systolic function in a contiguous 7.3 cm^2^ patch of the basal RV freewall combining transverse (38%), longitudinal (35%) and circumferential (27%) contraction and coinciding with the four‐chamber view. In the test cohort, all single‐point surrogates correlated with RV ejection fraction (p < 0.010), but correlation (R) was higher for SPM‐O (R = 0.44, p < 0.001) than TAPSE (R = 0.24, p < 0.001) and SFD (R = 0.22, p < 0.001), and non‐significantly higher than TAPSE‐F (R = 0.40, p < 0.001) and SFD‐F (R = 0.43, p < 0.001). SPM‐O explained more of the observed variance in RV ejection fraction (19%) and predicted it more accurately than any other 2D marker (median error 2.8 ml vs 3.6 ml, p < 0.001). We conclude that systolic motion of the basal RV freewall predicts global function more accurately than other 2D estimators. However, no markers summarise 3D contractile patterns, limiting their predictive accuracy.

## Introduction

Right ventricular (RV) function has prognostic value in acute 1, 2 and chronic 3, 4, 5, 6 cardiorespiratory disease and in the peri‐operative period 7, 8, 9, although rapid and accurate assessment is challenging due to the right ventricle's complex geometry and motion 10, 11. Several regional surrogate measures of global RV function have been investigated, most commonly targeting the contributions of longitudinal and transverse motion, because these are thought to predominate 12. In normal subjects, longitudinal shortening may account for the majority of RV function 13 and can be measured by tricuspid annular plane systolic excursion (TAPSE). However, TAPSE may be unreliable when assessed by less experienced operators 14 and in mild and moderate RV dysfunction 15. Prospective evaluations suggest TAPSE's prognostic value is limited 16, 17. Identifying the optimal two‐dimensional (2D) index of systolic function is difficult as some regions are easy to identify on imaging, some are influential on ventricular function and some are affected by dysfunction. These three areas do not necessarily coincide 18 and may change because of disease processes 19. Determining the individual contribution made to global function by the excursion of each point of the right ventricle requires a computational model of cardiac motion. Cardiac magnetic resonance (CMR) is considered the reference standard for RV volumetry and, recently, high‐resolution three‐dimensional cardiac magnetic resonance (3D‐CMR) has been used for accurate segmentation of whole‐heart anatomy 20, 21, 22. The variation in regional motion within a population can then be explored using atlas‐based segmentation techniques, where each subject's images are co‐registered 23, 24. We decided to apply these techniques in order to determine which area of the right ventricle best reflects global function in the general population, and tested its predictive performance against conventional indices such as TAPSE and septum‐freewall displacement (SFD).

## Methods

**Figure 1:**
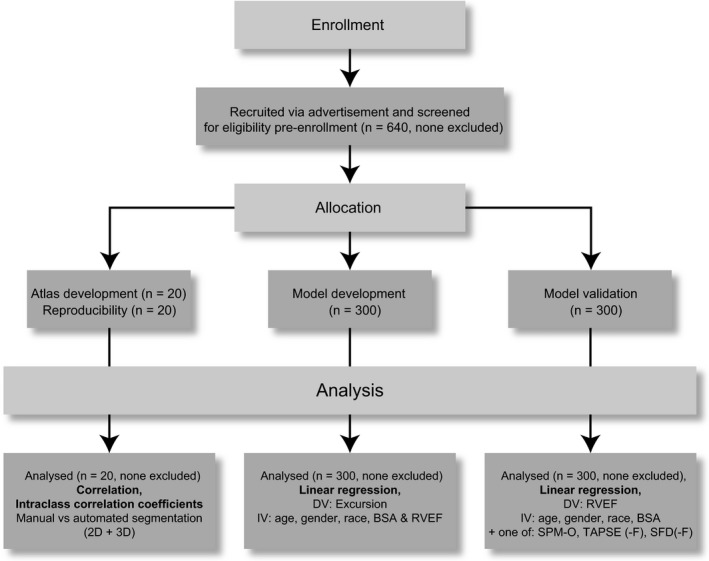
An overview of the study design. 2D, two‐dimensional; 3D, three‐dimensional; DV, dependent variable; IV, independent variable; BSA, body surface area; RVEF, right ventricular ejection fraction; SPM‐O, optimised single‐point marker; TAPSE(‐F), tricuspid annular plane systolic excursion (‐fractional); SFD(‐F), septum‐freewall displacement (‐fractional).

This study was conducted at a single centre and was approved by the Hospital's research ethics committee. Participants were recruited by advertisement and all gave written, informed consent. We did not study patients with cardiovascular disease or pregnancy, or those taking medication for hypertension, diabetes or hypercholesterolaemia. Standard published safety contraindications to MR imaging were applied 25. The study was divided into atlas development, model development, and model validation stages (Fig. 1). Images from 40 subjects were chosen at random: 20 were used to create a cardiac atlas (10 women, age range 24–59 years), 20 were imaged twice to assess reproducibility (9 women, age range 18–54 years). Images from the remaining 600 volunteers were divided equally into a model development group and a model validation group.

The CMR examinations were performed on a 1.5 T Philips Achieva system (Philips Medical Systems International, Best, the Netherlands). In order to capture the whole‐heart phenotype, a high‐spatial resolution, 3D balanced, steady‐state, free procession cine sequence was used that assesses the left and right ventricles in their entirety in a single breath‐hold (voxel size 2 × 2 × 4 mm reconstructed to 2 × 2 × 2 mm, 60 sections, flip angle 50°, bandwidth 1250 Hz.pixel^−1^, TE 1.5 ms, TR 3.0 ms, 20 cardiac phases, sensitivity encoding (SENSE) factor 2.0) 22. Conventional 2D cine imaging was also performed. Images were curated on an open‐source image database (MRIdb, Hammersmith Hospital Campus, Imperial College London, UK) 26.

Automated segmentation of the 3D‐CMR images was used to extract the myocardial shape and quantify phenotypic parameters. Segmentation was implemented using Matlab (MATLAB and Statistics Toolbox Release 2013b, The MathWorks Inc., Natick, MA, USA) and performed on a high‐performance server (Dell Technologies, Round Rock, TX, USA). The method uses prior knowledge by comparing each new set of images with a series of cardiac atlases where the anatomy has been manually defined 27. Each subject's dataset was co‐registered to a 3D spatial template created from an average cardiac shape to ensure that every point in the statistical model had the same anatomical correspondence throughout the population. Non‐rigid registration between cardiac phases was used to model RV motion 27. Mean unsupervised automated analysis time was 46 min per 3D sequence.

Segmentation accuracy was confirmed by visual inspection of the segmented images, by voxel concordancy with manual segmentation of the same subjects and by comparison of RV ejection fraction (RVEF) measured by two readers with 2 years’ cardiac MR experience using CMRtools (Cardiovascular Imaging Solutions, London, UK) with RVEF derived from the model. Segmentation reproducibility was confirmed using repeat imaging of the same subjects.

In model development, local function was assessed as the distance travelled in 3D space from end‐diastole to end‐systole by each of the 11,262 points representing the RV wall (‘excursion’). Global function was assessed by RVEF using a method previously described. In model validation, ventricular function was assessed by four single‐point measures in clinical use: TAPSE, fractional tricuspid annular plane systolic excursion (TAPSE‐F), SFD and fractional septum‐freewall displacement (SFD‐F) and their measurement followed published methods 14, 18. A fifth, novel measure was also taken, derived from the model development process described below. Data were analysed using R version 3.0.1 (R Development Core Team, Foundation for Statistical Computing, Vienna, Austria) 28.

Test–retest reproducibility was assessed using bias (mean and standard deviation) and intra‐class correlation coefficient (ICC) with a two‐way random model for absolute agreement 29. Regional excursion was assessed for association with global systolic function using pointwise bootstrapped linear regression adjusted for age, sex, race and body surface area (BSA). Data were centred and scaled before analysis. Significance was calculated using permutation testing (10,000 permutations) with correction for multiple testing by false‐discovery rate.

Adjacent points on the RV wall are likely to have highly correlated function (multicolinearity), increasing the risk of unreliable predictions in new datasets. Principal component analysis (PCA) was used to overcome this problem. ‘Principal components’ are orthogonal modes of variation seen in the data, from which the original dataset can be described with maximal economy. Being orthogonal, principal components are linearly uncorrelated with each other, and are therefore suitable variables for regression analysis and predictive models. The first, largest, principal component (PC1) was retained for subsequent regression. Only one component was retained to ensure estimations of the predictive strength of the 3D model were conservative, and to allow a direct comparison with other ‘single‐variable’ markers, such as TAPSE.

In model validation, each of the five 2D measures was tested by correlation, linear regression and accuracy of RVEF prediction. Correlation with RVEF was assessed using the Pearson product‐moment coefficient. Difference in correlation coefficients was assessed by a method described by Steiger 30. The association of each 2D measure with RVEF was assessed by linear regression with these variables, data were centred, scaled and coefficients bootstrapped (10,000 replications) with RVEF as the dependent variable and age, sex, race, BSA and the 2D measure of interest as independent variables (see also Supporting Information Table S1). Difference in regression models was assessed by treating the model type as an additional covariate. Right VEF prediction was tested using a leave‐one‐out analysis where RVEF for a single subject was predicted from the remaining 299 subjects and the process was repeated for all 300 validation subjects. Median absolute prediction errors were compared by Kruskal–Wallis rank sum test (χ^2^ statistic). A p value of < 0.05 was considered statistically significant.

## Results

Six hundred and forty adult volunteers successfully completed the imaging protocol. All datasets were used for analysis and there were no failures of the segmentation algorithm. Baseline characteristics of participants included in the study are shown in Table 1.

**Table 1:** Baseline characteristics of healthy volunteers (n = 600) included in the discovery and validation cohorts. Values are number (proportion) or median (IQR [range])

Characteristic	
Race	
African	18 (3%)
Afro‐Caribbean	30 (5%)
Asian	72 (12%)
Caucasian	450 (75%)
Chinese	12 (2%)
Other	18 (3%)
Sex; female	330 (55%)
Body surface area; m^2^	1.80 (1.69–1.95 [1.34–2.57])
Age; years	39.0 (29.5–47.6 [19.0–72.0])
Right ventricular ejection fraction; %	57 (52–62 [40–73])

No significant bias was detected between 3D automated, 3D manual and 2D manual volumetry of the right ventricle (bias + 8 ml.m^−2^_,_ limits of agreement: −6 to + 22 ml.m^−2^). Intraclass correlation coefficients for test–retest reliability of the model were 0.99 for 3D automated RV volume index and 0.93 for pointwise systolic excursion. Mean ± SD point distance from automated to manual segmentation was 1.46 ± 1.46 mm (95%CI: 1.46–1.47).

**Figure 2:**
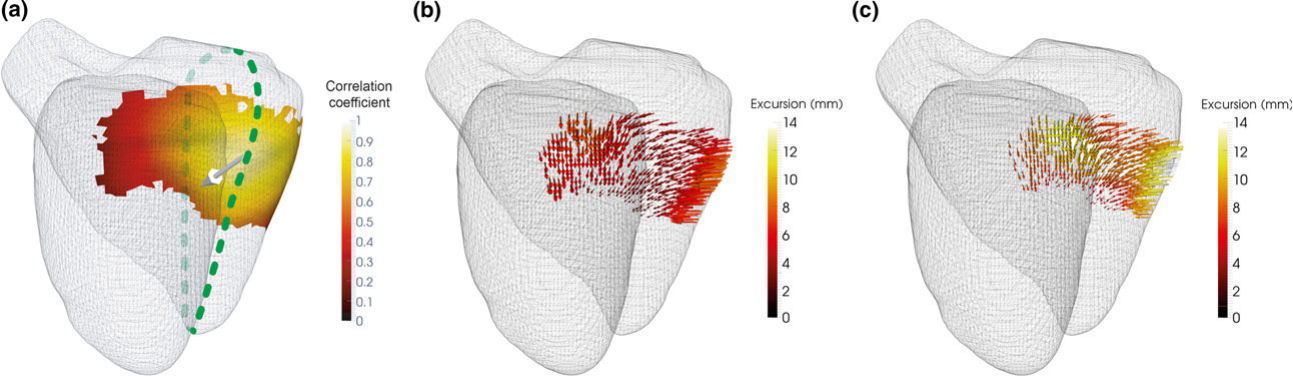
Regional function and principal component analysis. (a) The area where a significant association exists between regional excursion and right ventricular ejection fraction (RVEF) is shown by the coloured patch. The scale indicates the strength of correlation between patch function and patch function described by the first principal component. Overall direction of patch motion is indicated by the white arrow and comprises longitudinal, transverse and circumferential components. The intersection with the four‐chamber plane (dotted line) was used to identify the location of an Optimised Single‐Point Marker (SPM‐O) for subsequent validation. PCA of excursion within the patch in subjects with (b) low RVEF (42%) and (c) high RVEF (74%) reveals how patch function varies with global function. Increasing the number of principal components increases the accuracy of the model, but at the expense of including the influence of noise. The vertical dashed line indicates the cumulative variance accounted for by PC1 (43%).

Bootstrapped linear regression identified a distinct, contiguous 7.3 cm^2^ patch in the basal freewall where excursion was significantly associated with RVEF (Fig. 2). The patch was located between 4.1 cm and 6.9 cm from the apex and represented 9% of the total RV surface area. We used PCA to create independent modes of variation within the excursion of this patch. The PC1 accounted for 43% of the observed variance in excursion and was significantly correlated with RVEF (R = 0.34, p < 0.001) and associated with RVEF in regression (F = 40.4, p < 0.001), Table 2.

**Table 2:** The association of motion in the identified patch with right ventricular ejection fraction (RVEF) as assessed by correlation and linear regression. In linear regression, variables associated with altered right ventricular ejection fraction are controlled for

	Comparison	Test statistic		p value
Correlation	RVEF ~ PC1		R = 0.34	< 0.001
Regression	RVEF ~ PC1 + Age + Sex + Race + BSA	PC1	F = 40.4	< 0.001
		Age	F = 17.9	< 0.001
		Sex	F = 8.8	0.003
		Race	F = 0.6	0.450
		BSA	F = 0.04	0.850

PC1, first principal component; BSA, body surface area.

Freewall function increased from apex to base and this was mostly due to an increase in longitudinal function (see also Supporting Information Figure S1). Patch function consisted of longitudinal contraction (35% of patch function, median (IQR [range]) excursion 8.9 mm (7.2–10.9 [5.7–13.2]), transverse contraction (38%, 9.7 mm, 8.5–11.1 [7.6–12.3]) and circumferential contraction (27%, 6.8 mm, 6.7–7.0 [6.6–7.0]).

**Figure 3:**
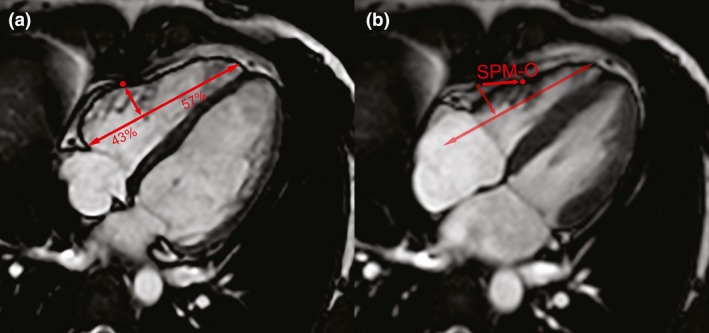
Extrapolation of computational model to manual marker of systolic function. The optimised single‐point marker (SPM‐O) is found by measuring displacement of a point from (a) end‐diastole to (b) end‐systole, in the plane perpendicular to a line between the lateral tricuspid annulus and the right ventricular apex, 43% of the way along.

To translate the automated computational findings to standard 2D analysis planes, a manual optimised single‐point marker (SPM‐O) measurement was derived by identifying a sub‐group of voxels within the patch coinciding with conventional scanning planes. Excursion values in the patch were reconstructed using PC1 and correlated with the original excursion value. The highest correlating voxel (R = 0.9) coinciding with a scanning plane was identified at 43% of the distance from base to apex, in the basal freewall of the four‐chamber plane. The displacement of this point from end‐diastole to end‐systole in millimetres was recorded manually from the four‐chamber plane (Fig. 3).

Right VEF, SPM‐O, and four validated markers of RV function (TAPSE, TAPSE‐F, SFD, SFD‐F) were evaluated prospectively in 300 subjects. All markers were significantly correlated with RVEF (Table 3). The SPM‐O had the highest correlation with RVEF (R = 0.44), which was significantly higher than that of TAPSE (p = 0.002) and SFD (p < 0.001), and non‐significantly higher than TAPSE‐F (p = 0.500) and SFD‐F (p = 0.860).

**Table 3:** The correlation of right ventricular function as assessed by two‐dimensional single‐point markers and by global, three‐dimensional volumetry

	Correlation coefficient	p value^a^	p value^b^
SPM‐O	0.44	< 0.001	–
SFD‐F	0.43	< 0.001	0.860
TAPSE‐F	0.40	< 0.001	0.500
TAPSE	0.24	< 0.001	0.002
SFD	0.22	< 0.001	< 0.001

SPM‐O, optimised single‐point marker; SFD‐F, septum‐freewall displacement (‐fractional); TAPSE‐F; tricuspid annular plane systolic excursion (‐fractional).

a Significance of correlation with RVEF.

b Significance of correlation difference compared with that of SPM‐O and right ventricular ejection fraction.

**Figure 4:**
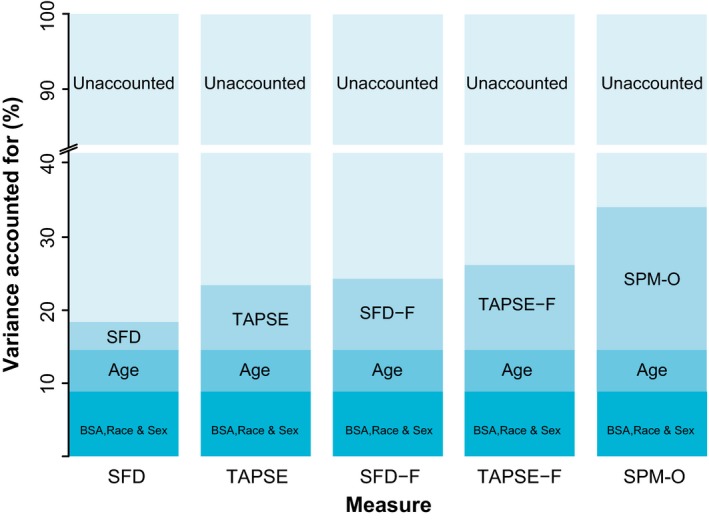
Proportion of variance in observed right ventricular ejection fraction accounted for by regression covariates. SFD(‐F), septum‐freewall displacement (‐fractional); TAPSE(‐F), tricuspid annular plane systolic excursion (‐fractional); SPM‐O, statistically optimised point marker.

In regression, SPM‐O accounted for the highest proportion of variance seen (19%) although differences between median (IQR [range]) were non‐significant (see also Supporting Information Table S2, Fig. 4). Leave‐one‐out analysis found that the median (IQR [range]) absolute error in the prediction of RVEF was significantly smaller for SPM‐O 2.8 (0.89–5.35 [0.00–18.75]) ml than other markers (p = 0.003, see also Supporting Information Table S3 and Figure S2).

## Discussion

Several parameters have been proposed to estimate global RV function, but this is the first report of a systematic computational approach to search for the optimal measurement. Using 3D‐CMR, we found that systolic displacement of the basal RV freewall is the strongest predictor of global function, outperforming TAPSE and other related indices in predicting RVEF. However, the 3D contractile pattern of the right ventricle limits the accuracy of estimators derived from 2D imaging planes. Clinicians managing patients at risk of RV dysfunction should be aware of these limitations in their assessments.

Right ventricular dysfunction is increasingly recognised as a significant predictor of morbidity and mortality in patients in the acute 1, 2, chronic 3, 4, 5, 6 and peri‐operative 7, 8, 9 settings. The complexity of RV function has prompted efforts to summarise function by markers of longitudinal 14, 15, 18 and transverse 31 function, though these markers have weaknesses 17, 32. Measures which combine both, such as fractional area change, appear to perform best but rely on accurate endocardial delineation and require suitable analysis software. These indices of RV function are derived from standard 2D imaging planes despite evidence that RV myocardial architecture is oblique to these planes, incorporates 3D contraction patterns and shows regional variation 33, 34. Furthermore, global function is affected by age, sex, race, BSA and interactions between these factors 35, 36, 37.

Our data show that the complex 3D contractile pattern of the right ventricle can be accurately assessed using CMR coupled with segmentation techniques, allowing consistent comparisons to be made between anatomical points across a population. This method allows modelling of the 3D contractile pattern of the right ventricle, which originates from its layered architecture with deep, longitudinal myofibrils 11 contributing to long‐axis excursion 38, 39 and superficial myofibrils contributing to transverse and circumferential contraction 11. Applying these approaches to a cohort of healthy subjects allows the contributions of these components to be assessed in the normal ventricle 18, 19. We suggest that although these contributions may vary in disease processes, and particularly in response to elevated RV pressure 19, 40, 41, 42, they are still useful for clinicians involved in the assessment of the right ventricle.

Principal component analysis was used to summarise wall motion as a single linear component, allowing translation to a conventional image plane and comparison with other single‐point excursion measures. This process retained the optimised balance of transverse and longitudinal contraction, reflected by SPM‐O's superior performance in comparison with other point markers. However, quantifying excursion in the long‐axis plane fails to account for circumferential contraction, which may explain why all 2D single‐point assessment methods have only a modest relationship with RVEF in the healthy population, even when optimised. The clinical value of this study is the finding that global RV function is most strongly associated with function in the basal RV freewall, but markers of function confined to two dimensions, although convenient to use and easily translated across imaging modalities, may fail to identify dysfunction orthogonal to the imaging plane.

Our study has several limitations. Work was confined to healthy volunteers with a presumed low incidence of regional wall motion abnormalities which are known to impair the performance of regional markers of global function 43. Regional markers based on excursion take no account of abnormal diastolic function or ventricular coordination, which may exist without systolic dysfunction 44. We evaluated this marker's performance using CMR in order to optimise global assessment so its reproducibility and performance in other imaging modalities and patient groups requires further investigation. Lastly, we have assumed that a linear relationship exists between clinical factors (age, race, sex, BSA), local function (as indicated by single‐point markers) and global function (as indicated by RVEF). Although this allows easy comparison of markers, we acknowledge that non‐linear relationships may exist between single‐point markers and clinical factors 45.

In conclusion, using 3D‐CMR with computational analysis allows a comprehensive understanding of how each component of RV motion contributes to pump function. Systolic excursion of the basal RV freewall is a better predictor of RVEF than other established parameters, including TAPSE, as it assesses both longitudinal and transverse contraction. This finding has implications for how global function is estimated using conventional 2D imaging.

## Supporting information


**Table S1.** Overview of model analysis methods in the discovery and validation cohorts.
**Table S2.** Regression parameters in the assessment of right ventricular function by two‐dimensional single‐point markers and by global, three‐dimensional ejection fraction.
**Table S3.** Leave‐one‐out analysis of SPM‐O and other two‐dimensional markers. N = 300.
**Figure S1.** Motion characteristics of the RV freewall. A plot to show the relative contribution of circumferential, longitudinal and transverse excursion within the right ventricle from apex to base (in a plane corresponding to the four‐chamber view). The dashed lines mark the zone in the freewall where global function (right ventricular ejection fraction) and local function (excursion) are significantly associated (4.1–6.9 cm, P < 0.05).
**Figure S2.** Prediction of RVEF in model validation cohort. RVEF, right ventricular ejection fraction; SFD(‐F), septum‐freewall displacement (‐fractional); TAPSE(‐F), tricuspid annular plane systolic excursion (‐fractional); SPM‐O, optimised single‐point marker.

## References

[1] Coutance G , Cauderlier E , Ehtisham J , Hamon M , Hamon M . The prognostic value of markers of right ventricular dysfunction in pulmonary embolism: a meta‐analysis. Critical Care 2011; 15: R103. 21443777 10.1186/cc10119 PMC3219376

[2] Zochios V , Parhar K , Tunnicliffe W , Roscoe A , Gao F . The Right Ventricle in ARDS. Chest 2017; 152: 181–93. 28267435 10.1016/j.chest.2017.02.019

[3] Dawes TJW , Cai J , Quinlan M , et al. Fractal analysis of right ventricular trabeculae in pulmonary hypertension. Radiology 2018; 288: 386–95. 29869959 10.1148/radiol.2018172821

[4] van de Veerdonk MC , Kind T , Marcus JT , et al. Progressive right ventricular dysfunction in patients with pulmonary arterial hypertension responding to therapy. Journal of the American College of Cardiology 2011; 58: 2511–19. 22133851 10.1016/j.jacc.2011.06.068

[5] Haddad F , Denault AY , Couture P , et al. Right ventricular myocardial performance index predicts perioperative mortality or circulatory failure in high‐risk valvular surgery. Journal of the American Society of Echocardiography 2007; 20: 1065–72. 17566702 10.1016/j.echo.2007.02.017

[6] Dawes TJW , de Marvao A , Shi W , et al. Machine learning of three‐dimensional right ventricular motion enables outcome prediction in pulmonary hypertension: a cardiac MR imaging study. Radiology 2017; 283: 381–90. 28092203 10.1148/radiol.2016161315 PMC5398374

[7] Peyrou J , Chauvel C , Pathak A , Simon M , Dehant P , Abergel E . Preoperative right ventricular dysfunction is a strong predictor of 3 years survival after cardiac surgery. Clinical Research in Cardiology 2017; 106: 734–42. 28409231 10.1007/s00392-017-1117-y

[8] Kaul TK , Fields BL . Postoperative acute refractory right ventricular failure: incidence, pathogenesis, management and prognosis. Cardiovascular Surgery 2000; 8: 1–9. 10.1177/096721090000800101 10661697

[9] Haddad F , Doyle R , Murphy DJ , Hunt SA . Right ventricular function in cardiovascular disease, part II: pathophysiology, clinical importance, and management of right ventricular failure. Circulation 2008; 117: 1717–31. 18378625 10.1161/CIRCULATIONAHA.107.653584

[10] Dell'Italia LJ . The right ventricle: anatomy, physiology, and clinical importance. Current Problems in Cardiology 1991; 16: 653–720. 1748012 10.1016/0146-2806(91)90009-y

[11] Ho S , Nihoyannopoulos P . Anatomy, echocardiography and normal right ventricular dimensions. Heart 2006; 92: i2–i13. 16543598 10.1136/hrt.2005.077875 PMC1860731

[12] Haddad F , Hunt SA , Rosenthal DN , Murphy DJ . Right ventricular function in cardiovascular disease, part I: anatomy, physiology, aging, and functional assessment of the right ventricle. Circulation 2008; 117: 1436–48. 18347220 10.1161/CIRCULATIONAHA.107.653576

[13] Brown SB , Raina A , Katz D , Szerlip M , Wiegers SE , Forfia PR . Longitudinal shortening accounts for the majority of right ventricular contraction and improves after pulmonary vasodilator therapy in normal subjects and patients with pulmonary arterial hypertension. Chest 2011; 140: 27–33. 21106653 10.1378/chest.10-1136

[14] Caudron J , Fares J , Vivier PH , Lefebvre V , Petitjean C , Dacher JN . Diagnostic accuracy and variability of three semi‐quantitative methods for assessing right ventricular systolic function from cardiac MRI in patients with acquired heart disease. European Radiology 2011; 21: 2111–20. 21614615 10.1007/s00330-011-2152-0 PMC3369832

[15] Nijveldt R , Germans T , McCann GP , Beek AM , van Rossum AC . Semi‐quantitative assessment of right ventricular function in comparison to a 3D volumetric approach: a cardiovascular magnetic resonance study. European Radiology 2008; 18: 2399–405. 18523785 10.1007/s00330-008-1017-7

[16] Weekes AJ , Johnson AK , Troha D , Thacker G , Chanler‐Berat J , Runyon M . Prognostic Value of Right Ventricular Dysfunction Markers for Serious Adverse Events in Acute Normotensive Pulmonary Embolism. Journal of Emergency Medicine 2017; 52: 137–50. 27751702 10.1016/j.jemermed.2016.09.002

[17] Biteker FS , Basaran O , Dogan V , Caylak SD , Yildirim B , Sozen H . Prognostic value of transthoracic echocardiography and biomarkers of cardiac dysfunction in community‐acquired pneumonia. Clinical Microbiology and Infection 2016; 22: e1–e6. 27596535 10.1016/j.cmi.2016.08.016

[18] Kind T , Mauritz GJ , Marcus JT , van de Veerdonk M , Westerhof N , Vonk‐Noordegraaf A . Right ventricular ejection fraction is better reflected by transverse rather than longitudinal wall motion in pulmonary hypertension. Journal of Cardiovascular Magnetic Resonance 2010; 12: 35. 20525337 10.1186/1532-429X-12-35 PMC2901360

[19] Pettersen E , Helle‐Valle T , Edvardsen T , et al. Contraction pattern of the systemic right ventricle shift from longitudinal to circumferential shortening and absent global ventricular torsion. Journal of the American College of Cardiology 2007; 49: 2450–6. 17599609 10.1016/j.jacc.2007.02.062

[20] Jenkins C , Bricknell K , Chan J , Hanekom L , Marwick TH . Comparison of two‐ and three‐dimensional echocardiography with sequential magnetic resonance imaging for evaluating left ventricular volume and ejection fraction over time in patients with healed myocardial infarction. American Journal of Cardiology 2007; 99: 300–6. 17261386 10.1016/j.amjcard.2006.08.026

[21] Lewandowski AJ , Augustine D , Lamata P , et al. Preterm heart in adult life: cardiovascular magnetic resonance reveals distinct differences in left ventricular mass, geometry, and function. Circulation 2013; 127: 197–206. 23224059 10.1161/CIRCULATIONAHA.112.126920

[22] de Marvao A , Dawes TJ , Shi W , et al. Population‐based studies of myocardial hypertrophy: high resolution cardiovascular magnetic resonance atlases improve statistical power. Journal of Cardiovascular Magnetic Resonance 2014; 16: 16. 24490638 10.1186/1532-429X-16-16 PMC3914701

[23] Fonseca CG , Backhaus M , Bluemke DA , et al. The Cardiac Atlas Project–an imaging database for computational modeling and statistical atlases of the heart. Bioinformatics 2011; 27: 2288–95. 21737439 10.1093/bioinformatics/btr360 PMC3150036

[24] Young AA , Frangi AF . Computational cardiac atlases: from patient to population and back. Experimental Physiology 2009; 94: 578–96. 19098087 10.1113/expphysiol.2008.044081

[25] Shellock FG . Reference manual for magnetic resonance safety, implants, and devices. Playa Del Rey, CA: Biomedical Research Publishing Group, 2017.

[26] Woodbridge M , Fagiolo G , O'Regan DP . MRIdb: medical image management for biobank research. Journal of Digital Imaging 2013; 26: 886–90. 23619930 10.1007/s10278-013-9604-9 PMC3782593

[27] Bai W , Shi W , O'Regan DP , et al. A probabilistic patch‐based label fusion model for multi‐atlas segmentation with registration refinement: application to cardiac MR images. IEEE Transactions on Medical Imaging 2013; 32: 1302–15. 23568495 10.1109/TMI.2013.2256922

[28] R Development Core Team . R: A language and environment for statistical computing. R Foundation for Statistical Computing. 2008 http://www.R-project.org. (accessed 04/10/2018).

[29] Bland JM , Altman DG . Measurement error and correlation coefficients. British Medical Journal 1996; 313: 41–2. 8664775 10.1136/bmj.313.7048.41 PMC2351452

[30] Steiger J . Tests for comparing elements of a correlation matrix. Psychological Bulletin 1980; 87: 245–51.

[31] Rushmer R . Cardiovascular dynamics, 4th edn Philadelphia, PA: W.B.Saunders, 1976.

[32] Schmid E , Hilberath JN , Blumenstock G , et al. Tricuspid annular plane systolic excursion (TAPSE) predicts poor outcome in patients undergoing acute pulmonary embolectomy. Heart Lung Vessel 2015; 7: 151–8. 26157741 PMC4476769

[33] Buckberg G , Mahajan A , Saleh S , Hoffman JI , Coghlan C . Structure and function relationships of the helical ventricular myocardial band. Journal of Thoracic and Cardiovascular Surgery 2008; 136: 578–589.e11. 18805255 10.1016/j.jtcvs.2007.10.088

[34] Hristov N , Liakopoulos OJ , Buckberg GD , Trummer G . Septal structure and function relationships parallel the left ventricular free wall ascending and descending segments of the helical heart. European Journal of Cardio‐Thoracic Surgery 2006; 29(Suppl. 1): S115–S125. 16564184 10.1016/j.ejcts.2006.02.041

[35] Maceira AM , Prasad SK , Khan M , Pennell DJ . Reference right ventricular systolic and diastolic function normalized to age, gender and body surface area from steady‐state free precession cardiovascular magnetic resonance. European Heart Journal 2006; 27: 2879–88. 17088316 10.1093/eurheartj/ehl336

[36] Maffessanti F , Muraru D , Esposito R , et al. Age‐, body size‐, and sex‐specific reference values for right ventricular volumes and ejection fraction by three‐dimensional echocardiography: a multicenter echocardiographic study in 507 healthy volunteers. Circulation. Cardiovascular Imaging 2013; 6: 700–10. 23811752 10.1161/CIRCIMAGING.113.000706

[37] Kawut SM , Lima JA , Barr RG , et al. Sex and race differences in right ventricular structure and function: the multi‐ethnic study of atherosclerosis‐right ventricle study. Circulation 2011; 123: 2542–51. 21646505 10.1161/CIRCULATIONAHA.110.985515 PMC3111939

[38] Petitjean C , Rougon N , Cluzel P . Assessment of myocardial function: a review of quantification methods and results using tagged MRI. Journal of Cardiovascular Magnetic Resonance 2005; 7: 501–16. 15881535 10.1081/jcmr-200053610

[39] Ghio S , Recusani F , Klersy C , et al. Prognostic usefulness of the tricuspid annular plane systolic excursion in patients with congestive heart failure secondary to idiopathic or ischemic dilated cardiomyopathy. American Journal of Cardiology 2000; 85: 837–42. 10758923 10.1016/s0002-9149(99)00877-2

[40] Leary PJ , Kurtz CE , Hough CL , Waiss MP , Ralph DD , Sheehan FH . Three‐dimensional analysis of right ventricular shape and function in pulmonary hypertension. Pulmonary Circulation 2012; 2: 34–40. 22558518 10.4103/2045-8932.94828 PMC3342747

[41] Haber I , Metaxas DN , Geva T , Axel L . Three‐dimensional systolic kinematics of the right ventricle. American Journal of Physiology. Heart and Circulatory Physiology 2005; 289: H1826–H1833. 15964922 10.1152/ajpheart.00442.2005

[42] Sakuma M , Ishigaki H , Komaki K , et al. Right ventricular ejection function assessed by cineangiography–Importance of bellows action. Circulation Journal 2002; 66: 605–9. 12074282 10.1253/circj.66.605

[43] Morcos P , Vick GW , Sahn DJ , Jerosch‐Herold M , Shurman A , Sheehan FH . Correlation of right ventricular ejection fraction and tricuspid annular plane systolic excursion in tetralogy of Fallot by magnetic resonance imaging. International Journal of Cardiovascular Imaging 2009; 25: 263–70. 19048388 10.1007/s10554-008-9387-0

[44] Dawes TJ , Gandhi A , de Marvao A , et al. Pulmonary artery stiffness is independently associated with right ventricular mass and function: a cardiac MR imaging study. Radiology 2016; 280: 398–404. 26909648 10.1148/radiol.2016151527 PMC4976460

[45] Koestenberger M , Nagel B , Ravekes W , et al. Left ventricular long‐axis function: reference values of the mitral annular plane systolic excursion in 558 healthy children and calculation of z‐score values. American Heart Journal 2012; 164: 125–31. 22795292 10.1016/j.ahj.2012.05.004

