# Supplementary material for: Identifying the optimal regional predictor of right ventricular global function: a high‐resolution three‐dimensional cardiac magnetic resonance study
Source: Anaesthesia. 2018 Nov 14;74(3):312–20. doi: 10.1111/anae.14494 (PMC6767156; doi:10.1111/anae.14494)
Supplement: Supplementary file 1 — Table S1. Overview of model analysis methods in the discovery and validation cohorts. Table S2. Regression parameters in the assessment of right ventricular function by two‐dimensional single‐point markers and by global, three‐dimensional ejection fraction. Table S3. Leave‐one‐out analysis of SPM‐O and other two‐dimensional markers. N = 300. Figure S1. Motion characteristics of the RV freewall. A plot to show the relative contribution of circumferential, longitudinal and transverse excursion within the right ventricle from apex to base (in a plane corresponding to the four‐chamber view). The dashed lines mark the zone in the freewall where global function (right ventricular ejection fraction) and local function (excursion) are significantly associated (4.1–6.9 cm, P < 0.05). Figure S2. Prediction of RVEF in model validation cohort. RVEF, right ventricular ejection fraction; SFD(‐F), septum‐freewall displacement (‐fractional); TAPSE(‐F), tricuspid annular plane systolic excursion (‐fractional); SPM‐O, optimised single‐point marker. [file ANAE-74-312-s001.docx]

# 21410 - SUPPLEMENTARY MATERIAL

**Table 1.** Overview of model analysis methods in the discovery and validation cohorts.

**Table 2.** Regression parameters in the assessment of right ventricular function by two-dimensional single-point markers and by global, three-dimensional ejection fraction.

**Table 3.** Leave-one-out-analysis of SPM-O and other two-dimensional markers. N=300.

**Figure 1. Motion characteristics of the RV freewall**

A plot to show the relative contribution of circumferential, longitudinal and transverse excursion within the right ventricle from apex to base (in a plane corresponding to the four-chamber view). The dashed lines mark the zone in the freewall where global function (right ventricular ejection fraction) and local function (excursion) are significantly associated (4.1-6.9 cm, P<0.05).

**Figure 2. Prediction of RVEF in model validation cohort**

RVEF, right ventricular ejection fraction; SFD(-F), septum-freewall displacement (-fractional); TAPSE(-F), tricuspid annular plane systolic excursion (-fractional); SPM-O, optimised single point-marker.

**Table 1.** Overview of model analysis methods in the discovery and validation cohorts

| **Method** |  | **Correlation (RVEF vs…)** | **Linear Regression** |
| --- | --- | --- | --- |
| **Discovery** |  | PC1 | RVEF ~ Age + Sex + Race + BSA + … |
|  |  | SPM-O |  |
| **Validation** | Longitudinal function | TAPSE |  |
|  |  | TAPSE-F |  |
|  | Transverse function | SFD |  |
|  |  | SFD-F |  |

RVEF indicates right ventricular ejection fraction; PC1, first principal component; SPM-O, optimised single-point marker; TAPSE(-F), tricuspid annular plane systolic excursion (-fractional); SFD(-F), septum freewall displacement (-fractional); and BSA, body surface area.

**Table 2.** Regression parameters in the assessment of right ventricular function by two-dimensional single-point markers and by global, three-dimensional ejection fraction.

|  | **F** | **Variance (%)*** | **p-value†** |
| --- | --- | --- | --- |
| SPM-O | 86.4 | 19 | <0.001 |
| SFD-F | 37.6 | 10 | < 0.001 |
| TAPSE-F | 46.8 | 12 | < 0.001 |
| TAPSE | 34.4 | 9 | < 0.001 |
| SFD | 13.3 | 4 | < 0.001 |

*Variance in the observed data accounted for by this covariate (100 x sum of squares / total sum of squares)

† Significance of association with right ventricular ejection fraction

SPM-O indicates optimised single-point marker; TAPSE(-F), tricuspid annular plane systolic excursion (-fractional); and SFD(-F), septum freewall displacement (-fractional).

**Table 3**. Leave-one-out-analysis of SPM-O and other two-dimensional markers. N=300

|  | **Median absolute error* (mLs)** | **IQR (mLs)** | **Range** |
| --- | --- | --- | --- |
| SPM-O | 2.82 | 0.89 - 5.35 | 0.00 – 18.75 |
| SFD-F | 3.49 | 1.73 – 6.38 | 0.04 – 17.62 |
| TAPSE-F | 3.62 | 1.65 – 6.37 | 0.01 – 19.12 |
| TAPSE | 3.80 | 1.88 – 6.74 | 0.02 – 19.07 |
| SFD | 3.64 | 1.83 – 6.80 | 0.01 – 20.27 |

*Kruskal Wallis chi-squared = 16.2, df = 4, p-value = 0.003

SPM-O indicates optimised single-point marker; TAPSE(-F), tricuspid annular plane systolic excursion (-fractional); SFD(-F), septum freewall displacement (-fractional); and IQR, inter-quartile range.

**Supplementary Figure 1. Motion Characteristics of the RV Freewall**

A plot to show the relative contribution of circumferential, longitudinal and transverse excursion within the right ventricle from apex to base (in a plane corresponding to the four-chamber view). The dashed lines mark the zone in the freewall where global function (right ventricular ejection fraction) and local function (excursion) are significantly associated (4.1-6.9 cm, P<0.05).

**Supplementary Figure 2. Prediction of RVEF in Model Validation Cohort**

RVEF , right ventricular ejection fraction; SFD(-F), septum-freewall displacement (-fractional); TAPSE(-F), tricuspid annular plane systolic excursion (-fractional); SPM-O, optimised single point-marker.
